# Fruit Detectability Analysis for Different Camera Positions in Sweet-Pepper †

**DOI:** 10.3390/s140406032

**Published:** 2014-03-27

**Authors:** Jochen Hemming, Jos Ruizendaal, Jan Willem Hofstee, Eldert J. van Henten

**Affiliations:** 1 Wageningen UR Greenhouse Horticulture, P.O. Box 644, 6700 AP Wageningen, The Netherlands; E-Mail: eldert.vanhenten@wur.nl; 2 Farm Technology Group, Wageningen University, P.O. Box 317, 6700 AH Wageningen, The Netherlands; E-Mails: josruizendaal@hotmail.com (J.R.); janwillem.hofstee@wur.nl (J.W.H.)

**Keywords:** fruit visibility, sweet-pepper, occlusion, harvesting, robotics

## Abstract

For robotic harvesting of sweet-pepper fruits in greenhouses a sensor system is required to detect and localize the fruits on the plants. Due to the complex structure of the plant, most fruits are (partially) occluded when an image is taken from one viewpoint only. In this research the effect of multiple camera positions and viewing angles on fruit visibility and detectability was investigated. A recording device was built which allowed to place the camera under different azimuth and zenith angles and to move the camera horizontally along the crop row. Fourteen camera positions were chosen and the fruit visibility in the recorded images was manually determined for each position. For images taken from one position only with the criterion of maximum 50% occlusion per fruit, the fruit detectability (FD) was in no case higher than 69%. The best single positions were the front views and looking with a zenith angle of 60° upwards. The FD increased when a combination was made of multiple viewpoint positions. With a combination of five favourite positions the maximum FD was 90%.

## 1. Introduction

In the framework of the European FP7 project Clever Robots for Crops—CROPS [1] several highly configurable and modular demonstrators are currently under development for robotic harvesting of high value greenhouse vegetables and fruits in orchards. One of the key issues in automated fruit harvesting is the localization of the fruits on the plant by means of a sensor system. The desired situation is to detect and localize close to 100% of all ripe fruits on the plant. For robotic harvesting of sweet-pepper fruits in a greenhouse a computer vision system is a possible approach for fruit detection. Due to the structure of the production system, images could then be taken using a device that is placed between the plant rows. In many cases however, fruits will not be completely visible because leaves or branches will hang in front of fruits or individual fruits are not clearly visible because they are often growing closely together in clusters. Publications from [2] or [3] confirm that the main problem in automated fruit harvesting is the visibility of the fruits in the crop. However, little information about fruit visibility is available so far from literature, and only a few authors, for example [4,5], give quantitative numbers.

In other studies multiple camera viewpoints were also used to acquire images of the crop. In the research of [6] images of cucumber plants were taken with a linear displacement of 0.33 m. Thereby every plant was visible in three subsequent images. The plant shifted in the images and was seen from a different angle in every picture. With three different pictures of one plant, on some pictures the fruits were occluded while on another picture of the same plant, the fruit was totally visible. In this way more than 95% of the cucumbers were detected correctly. In a research on a cherry harvesting robot [7] different positions around the crop were studied to increase visibility. Images were taken from four different positions around the trunk of the plant. It was stated that 59% of the fruits were visible when all images were used. In research by [8] multiple positions around a citrus tree were investigated to determine the positions that were needed to get the highest fruit visibility. A combination of up to six views resulted in a significantly higher visibility.

In this research, the effect of multiple camera positions and viewing angles on fruit detectability in a sweet-pepper greenhouse crop was investigated. The objective was to determine the optimal camera position or combination of positions which yields the maximum visibility of the sweet-pepper fruits on the plant for the purpose of robotized harvesting. Little information can be found concerning the minimum visible fruit surface a computer vision system needs, to be able to detect and localize a fruit. According to [5] the segmentation for spherical objects (citrus) proves very efficient for spheres visible for more than 50% of their surface. To our own experience, fruits that are occluded for more than 50% are hard to localize with the precision required for robotic harvesting. Therefore most results on fruit detectability presented in this research are under the precondition that at least 50% of the fruit surface is visible from a certain viewpoint.

## 2. Materials and Methods

### 2.1. Recording Device

**Figure 1.**
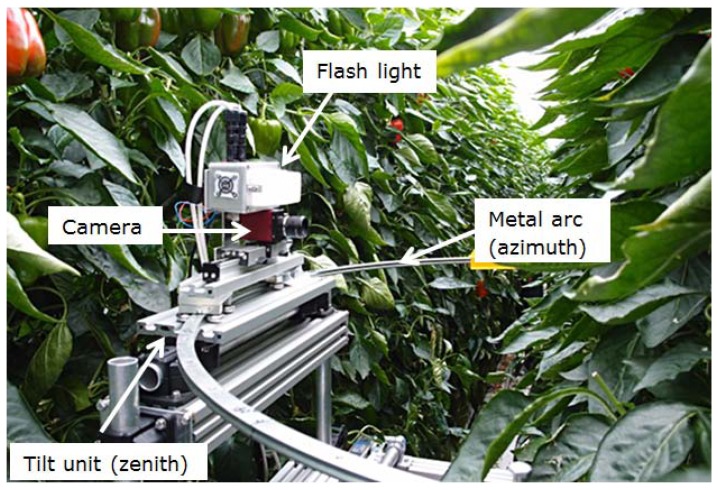
(**a**) Photo of the camera setup in the crop row, with azimuth = 90° and zenith = 90°.

A recording device was built which allowed placing the camera under different azimuth and zenith angles. The device was placed on a crop handling cart so it could easily be moved along the crop row as well (Figure 1). A 1/1.8 inch CCD colour camera (Stingray F201C, Allied Vision Technology, Stadtroda, Germany) was used for the recording. The camera was equipped with a low distortion wide angle lens with a focal length of 4.16 mm (Lensagon CMFA0420ND, Lensation, Karlsruhe, Germany). On a distance of 0.5 m the field of view covered a crop area of about 0.7 m by 0.5 m. This area was sufficient to capture the region where all ripe fruits were located. In the rare case a fruit on the plant was located outside the captured region it was not counted and not included in the analysis. The camera was mounted on a slide with a tilt unit which could be moved on a metal arc with a diameter of 1.0 m. Tilt unit and arc allowed setting the camera to different azimuth and zenith angles. Before acquiring images the recording device was positioned such that the recorded plant was located in the centre of the arc. The height of the recording device was changed for every plant to have the lowest coloured fruit in the middle of the image. Images were recorded with a resolution of 1624 × 1234 pixels. On top of the camera a flash light (MVS 5002 PerkinElmer, Groningen, The Netherlands) was mounted to ensure a defined illumination of the scene.

### 2.2. Terms and Definitions

**Figure 2.**
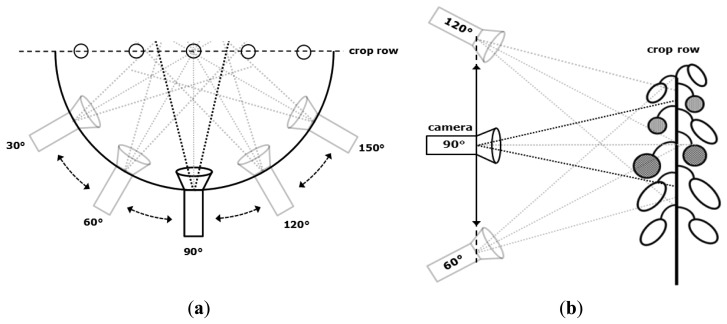
(**a**) Azimuth angles for the camera; (**b**) Zenith angles for the camera.

Throughout this paper the terms azimuth and zenith angle will be used to describe the position and orientation of the camera. Based on the acquired images, the fruit visibility and fruit detectability will be assessed. These terms are defined as follows:

Azimuth angle: a celestial coordinate system is used that describes the position and orientation of the camera in the horizontal plane. The reference point for this orientation is located at the intersection of the line of view of the camera and the crop row. Then, as illustrated in Figure 2a an azimuth of 90° defines to a camera position straight in front of the crop row.
Zenith angle: The zenith is a vector pointing up from a point of interest, perpendicular to the fundamental plane. In this case, the zenith is pointing up from the middle of the camera. As illustrated in Figure 2b the zenith angle is defined as the angle between the vector from the camera to the crop row and the zenith vector.
Fruit visibility (FV): The visible part of a fruit in an image expressed as a percentage of total fruit area which would be seen in an image without occlusion. Ranging from 0% visibility (not visible at all) to 100% visibility (completely visible).
  Fruit detectability (FD): The relative number of fruits (%) on a plant that is visible for at least a certain FV percentage. Example: with a FV threshold set to 40%, a fruit which is occluded for not more than 60% in an image will be counted as detected. In case of using several viewpoint combinations, as explained later, a fruit is counted as detected if its FV was above the set threshold in at least one of the images.

The sample standard deviation (SD) of the FD for a specific viewpoint position was calculated using Equation (1):

(1) $$SD=\sum_{i=1}^{n}\left(\frac{FD_{i}-\bar{FD}}{n-1}\right)$$

where:

*FD_i_* is the FD of plant *i*
$\bar{FD}$ is the average FD
*n* = total number of plants.

### 2.3. Positioning of the Camera During the Image Acquisition Sessions and Collection of Ground Truth Data

**Figure 3.**
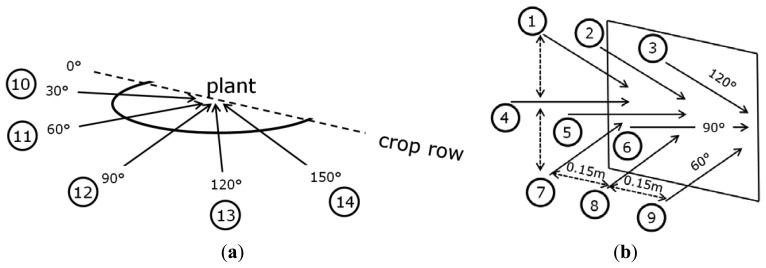
(**a**) Label numbers for the different azimuth positions from which images were taken; (**b**) Additional label numbers for camera positions used in the second and third measurement session. The square represents the crop canopy.

During the image acquisition sessions, the camera was placed in several positions and orientations with respect to the crop using the recording device. Position and orientation were varied in three different ways by changing: the azimuth angle, the zenith angle and the horizontal position with respect to the crop row (Figures 2 and 3).

In all three measurement sessions the camera was first set to a zenith of 90° and then placed in five different azimuth angles, namely 30°, 60°, 90°, 120° and 150°. In Figure 3 these positions are labelled 10 to 14. For the second and third measurement session the zenith angle was additionally varied and set to angles of 60°, 90° and 120°. For every zenith angle three images were taken with a horizontal displacement of 0.15 m each. The scene was chosen such that the plants and their fruits were visible in all the three images. These positions are labelled 1,2,3; 4,5,6 and 7,8,9 (Figure 3). Label 5 and 12 account thus for the same position but were numbered separately due to the experimental setup. Following this procedure, images were taken from five different viewpoints of every plant in the first session, and from 14 viewpoints in the second and third session. In total 330 images were taken of 30 plants during the three image acquisition sessions as indicated in Table 1.

For every session, ground truth data was collected to be able to evaluate the results for the fruits visible in the recorded images. The number and positions of individual fruits on the plants were registered in the greenhouse. The purpose of this registration was to be able to later also account for fruits which are completely occluded (e.g., by leaves) in the recorded images. The FV of every registered fruit was estimated by human offline review of the acquired images based on proficiency. The human observer assigned to each fruit a FV value of one of 0%, 5%, 10%, 20%, 30%, 40%, 50%, 60%, 70%, 80%, 90% or 100%. No automated computer vision and image analysis procedure was carried out for estimating the FV. This was done for all recorded viewpoints. Reference scenes of the viewpoints with all fruits 100% visible could not be recorded as this would have had major impact on the crop caused by removing leaves. To assess the detectability of the fruits, the FD was calculated. During the detectability assessment, thresholds in the range of 5% to 100% minimum FV were used. In addition to this, for each scene all unique combinations of viewpoints were derived to identify the impact of multiple viewpoints on fruit detectability. For the five azimuth angles 31 unique combinations are possible (Equation (2)):

(2) $$\left(\begin{array}{c} 5\\ 1 \end{array}\right)+\left(\begin{array}{c} 5\\ 2 \end{array}\right)+\left(\begin{array}{c} 5\\ 3 \end{array}\right)+\left(\begin{array}{c} 5\\ 4 \end{array}\right)+\left(\begin{array}{c} 5\\ 5 \end{array}\right)=31$$

The nine positions (labelled 1–9) result in 511 possible combinations. With azimuth angles, zenith angles and different horizontal displacements combined 16,383 unique combinations are possible. For each combination of images the FD was calculated.

### 2.4. Crop

Images were taken of greenhouse crops of yellow and red cultivars of sweet pepper (*Capsicum annum*) at two different commercial growers in The Netherlands. Besides the difference in colour, the crops also differed in density. The red species had more leaves and the plants were in general more voluminous than the yellow cultivar. As it is good practice in a commercial crop, consecutive leaves or side branches from neighbouring plants overlapped to a certain degree, average plant distance in the row was about 0.2 m. The plants and neighbouring plants were not manipulated or shifted before image recording but recorded as found at the commercial growers. The free space between the rows was limited and resulted on average in a distance of 0.5 m between the sensor and the canopy. Measurement session 2 and 3 were recorded in the same greenhouse with the same cultivar at two different moments of the season. For sweet-peppers grown in Dutch greenhouses harvest takes place weekly and there are mature fruits available from March to October. There was no difference in fruit maturity levels at the different measurement sessions, but as the plants are continuously developing, the number of fruits per plant changed during the season. This number was also affected by previous harvest operations on the same plant. Table 1 gives an overview of the crop characteristics. The total number of fruits given is for the number of fruits for all plants together.

## 3. Results

### 3.1. Azimuth Angles

**Figure 4.**
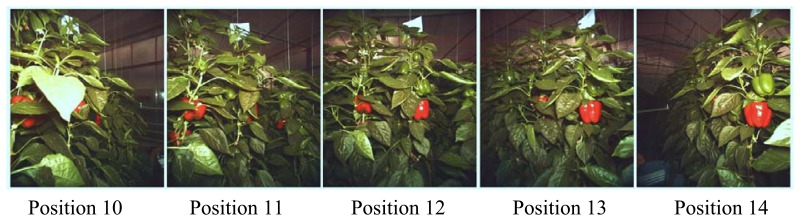
Example images from the same plant taken from different camera viewpoints.

Figure 4 shows example images taken of the same plant from different azimuth angles. At position 10 and 11 the red coloured fruit on the target plant in the centre of the image is almost completely occluded. At position 12 the FV of this fruit is more than 50%. At position 13 and 14 the same fruit is not occluded and thus has a FV of 100%.

**Figure 5.**
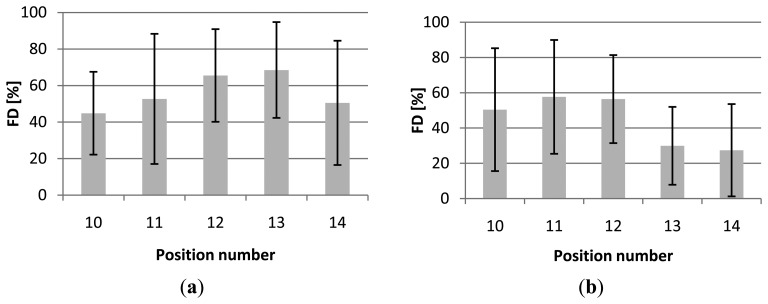
(**a**) Average FD and standard deviation (SD) for different azimuth angles, for cultivar Helsinki (first session); (**b**) Average FD and standard deviation (SD) for different azimuth angles, cultivar Nagano (third session).

Applying a FV threshold of 50%, the average FD ranged from 45% to 69% for cultivar Helsinki (first session), from 20% to 31% for the second session (cultivar Nagano) and from 27% to 58% for the third session (cultivar Nagano). Figure 5 gives the detailed results per viewpoint position for the first and the third session including the standard deviation (SD) for every viewpoint.

The average FV at the two recording dates differed considerably along the season for cultivar Nagano, namely 20% during the second session and 38% during the third session. For a high number of recorded plants the most extreme viewpoint positions (10 and 14) show the lowest FD, however concerning the average values this is only true for the first session (Figure 5b). The positions with the highest FD with respect to the azimuth angles are for session 1 position 13 (FD = 69%), for session 2 position 12 and 13 (both FD = 31%) and for session 3 position 11 (FD = 58%). However, this does not mean that positions 10 and 14 are not useful. When combining positions (Section 3.3) for improved visibility, these positions can give valuable information about the location of fruits which were not visible in the other positions. The high SD in FD indicates that the average fruit detectability differs very much per plant. The large SD values in Figure 5 also show that there are no significant differences in the FD for the different camera azimuth angles. Both cultivars (Helsinki and Nagano) with their different crop properties as described in Section 2.4, show a similar level of SD.

### 3.2. Zenith Angles

The measurements for the different zenith angles and horizontal displacements were only performed for the red sweet-peppers during the second and third measurement session. Also in this case, to calculate the FD, the threshold for a sweet pepper to be counted as visible was set to a FV ≥ 50%. Positions 1, 2 and 3 show the lowest FD, with a minimum value of 3% for position 3. Position 3 shows a notable lower FD on the first measurement. In Table 2 these results are presented.

In the positions 1, 2 and 3 the images were taken with a zenith angle of 120°. With this angle the leaves were about perpendicular to the camera which resulted in occlusion of the fruits by leaves in these images. The FD for this zenith angle is significantly different (*p* = 0.05) from the zenith angles 60° and 90°. The covering of the fruits by the leaves from this position is probably a natural protection of the fruits by the crop for direct sunlight. The positions 4 to 9 all have a FD between 27% and 43% for the second session and between 39% and 65% for the third session. In these positions, images were taken with a zenith angle of 60° and 90°. They show most information about the location of the fruits. As for the azimuth angles the standard deviation indicates that the average fruit visibility differs very much per plant.

For all viewpoint positions the average FD shown in Table 2 was much higher in the third session than in the second session. The best single positions for the zenith angle experiments were the front views (position 5 for the second session and position 4 for the third session) and positioning the sensor in a zenith angle of 60° (positions 7, 8 and 9).

### 3.3. Combined Positions

**Figure 6.**
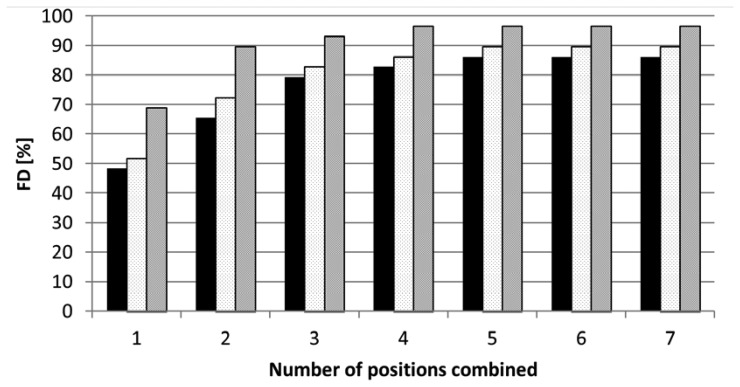
FD for combined positions and with different FV threshold values Results from the third session. FV threshold = 60% (black bars); 50% (white bars); 40% (grey bars).

As expected, the FD increases when a combination was made of multiple viewpoint positions. Figure 6 shows maximum values for FD for a number of positions combined for different FV thresholds. The maximum FDs shown in this figure is the maximum FD of all possible 16,383 unique combinations for the different azimuth angles, zenith angles and different horizontal displacements (see also Section 2.3).

For the 3rd measurement session a maximum FD of 86% was reached by combining four or five positions in case of a FV threshold value set to 50%. For every FV threshold value there were more possible combinations of positions which gave the maximum FD. The sets of combinations however were not all the same for the different measurement sessions. However, common sets of favourite viewpoint positions for all measurement sessions could be found. These values are given in Table 3. With a combination of these favourite five positions, the maximum FD was 90% with a FV threshold value set to 50%. This set of images consisted of two images with a zenith of 60° and an azimuth of 90° with a horizontal displacement of 0.15 m and three images with a zenith of 90° and an azimuth of 30°, 90° and 120°. The same set of five positions yielded the maximum FD in the second session, *i.e.*, 76%. In addition to this, Table 3 shows also the results for FV threshold values of 40% and 60%. Table 4 shows more details for different visibility thresholds. In case the FV threshold value was set to 10%, three camera positions only were needed to yield a FD of 93% during the second session and two positions only to yield a FD of 97% during the third session. The addition of more positions did not further increase the FD in those cases.

## 4. Discussion and Conclusions

The results of this study have shown big differences in the average FD of the different sessions. These could be due to the different cultivars, different fruit load and different plant maintenance actions the different growers applied. Session 2 and session 3 were recorded in the same greenhouse and this indicates that the obtained FD may be strongly influenced by a seasonal effect. Later in the season plants are taller and it can be speculated that the differences in FD are due to a changed plant structure. Also work published on sensor systems on other agricultural harvesting robots (*i.e.*, strawberries) confirm that the visibility of the target changes during the season [4].

Concerning the azimuth angles the initial expectation was that FD will be the highest from positioning the camera straight in front of the row (position 12) and that the FD will decrease with increasing viewing angle to the left and right. This, however, was not supported clearly by the data. The highest FD was still obtained at one of the positions with lower view angle (11 or 12 or 13) and decreased at the positions with higher view angle (10 and 14). However, as stated above, differences in azimuth angles are not statistically significant. At the extreme angled positions parts from neighbouring plants were interfering with the free line of view. However, the trend is in agreement with the results of the zenith angle experiments (Table 2). Also here the front positions (position 5 and 4) resulted in the highest FD. Moreover, it is remarkable that in the third session the FD for position 13 dropped whereas in session 1 (and also session 2, but data not shown) the FD for position 13 was high. Also, as shown in Figure 5b, the FD's for position 13 and 14 are much lower than position 10 and 11 what shows a not symmetrical decline of the FD from the centre viewpoint to the more angled viewpoints to both sides. One explanation could be an asymmetrical structure of the plant canopy along the plant row. Leaves of the plant might face to one side towards the light and therefore show different fruit visibility levels from different viewpoints.

Most of the experiments were carried out with a FV of 50%. At this point of the development it is not clear how much amount of fruit is needed for a sensor system to be sure it is detecting a fruit and future work should focus on that aspect. As stated in the introduction already, natural objects occluded by more than 50% will most likely be hard to localize with precision required for robotic harvesting For robotic harvesting, not only fruit detection but also fruit localization must take place. In addition to this a robotic end-effector to grip and detach the fruit will most likely require accurate data about the fruit pose. The determination of the fruit real world position and orientation will be a difficult task with only a small percentage of fruit surface visible.

In the presented study the ground truth FV percentage of a fruit was estimated by a human observer based on an image of the scene only. Due to the irregular shape and size of the fruits an accurate determination of the FV percentage remains impossible using that approach. To increase the accuracy two images should be taken from every position. One image of the natural scene and one image with all fruits 100% visible, for example by picking leaves. However, this would have had major impact on the future growth of the crop and as this study was carried at commercial growers it was opted to not do so. Moreover, this study was aiming on revealing the major effects of different viewpoints on the FD and small inaccuracies in estimating the FV are not expected to change the conclusions of this study.

For robotic harvesting of fruits, the detection and localization of the fruits on the plant is mandatory. For images taken from one position only with a FV threshold of 50%, the FD was in no case higher than 69% (position 13, Figure 5a), which is much lower than the desired 100%. Not surprisingly, combining multiple viewpoint positions enhanced the result. As described above the maximum FD was 90% (minimum 50% FV) or 97% (minimum 10% FV). As shown in Table 3 the maximum FD (minimum 50% FV) was reached with a combination of five positions, with the addition of more positions the FD did not increase. It is difficult to compare the results obtained to the results obtained by e.g., [5] or [6] because it is not clearly defined in those publications for what extend the fruit (surface) must be visible before counted as detected and because they dealt with different crops. However, the work presented confirms the results from these authors and also from [7] in such a way that multiple views of the target plant are needed to reach fruit visibility levels suited for robotic harvesting.

Next to the idea of using multiple viewpoints there are more possibilities to increase the visibility of fruits. Plant locations and densities could be adapted in the row. Modifications to the cultivation system can simplify the environment. By introducing the high-wire cropping system for cucumbers fruit occlusion was drastically reduced [6]. To study the effect of such modifications was not an objective of this paper but it emphasizes that the development of a robotic harvesting system has to take into account not only one but many different elements. Systems that increase visibility of the target objects by using an air blower as done for melon harvesting [9] or for leave detection in tomato [10] have been described in literature. For the specific task of robotic pepper harvesting there are a number of issues with blowing leaves. As the overall plant canopy is flexible, blowing could significantly and dynamically change the geometry of the scene. The determined fruit locations while blowing may not be accurate and static enough for a robotic system. Moreover, blowing a high volume of air through the canopy gives the risk of spreading pests and diseases from one plant to another plant and the growers dislike that idea.

In robotics and automation in general and for 3D mapping of scenes by range sensors in particular, the determination of the best view or viewpoint of a scene is a research area related to the work presented here. There are a number of algorithms developed to determine the “best” next view to take with a sensor (e.g., [11–13]). These algorithms are in general based on 3D sensors incrementally adding range data to a partial model until the entire object has been scanned. Even in the field of plant monitoring some work is published for determine positions for a 3D camera that offers a better view of a target leaf [14]. For future work it would be very interesting to investigate if these contributions can help to define camera positions for the presented application which yield in a maximum fruit detectability.

In conclusion, a recording device for sweet-pepper fruit localization used by an autonomous harvesting robot will largely increase its detection rate if it acquires images from multiple viewpoints of the same plant. The FD is strongly influenced by differences in plant structure and a seasonal effect.

A fruit localization system should be able to detect the presence of a fruit already when only a small part of the fruit surface is visible in the image. To reach a high percentage of harvested product, an additional sensor system mounted on the robotic arm might help. Such a sensor can enter the crop canopy to detect fruits not visible at all from the main path due to the heavy occlusion. However, taking images from several viewpoints will either be time consuming due to the repositioning of the sensor, resulting in lower cycle times for the harvest operation, or will increase the costs of the system because multiple sensors mounted at different viewpoints are needed. An economical trade-off must be found between the needed percentage of detected product and the available room for investment.

## Figures and Tables

**Table 1.** Crop characteristics during the measurements.

**Session**	**Recording date**	**Cultivar**	**Fruit colour**	**Crop height**	**Number of plants**	**Total number of fruits**
1	17 May 2011	Helsinki	Yellow	1.5 m	10	32
2	24 June 2011	Nagano	Red	2.4 m	10	29
3	7 September 2011	Nagano	Red	2.8 m	10	35

**Table 2.** Average FD and standard deviation (SD) for position 1–9 for the second and third session with a FV threshold of 50%.

**Position**	**FD [%] 2nd session**		**FD [%] 3rd session**	

	**Average**	**SD**	**Average**	**SD**
1	14 ^a^	19	26 ^a^	23
2	12 ^a^	15	40 ^a^	31
3	3 ^a^	8	24 ^a^	24
4	27 ^b^	31	65 ^a^	29
5	43 ^b^	20	56 ^a^	26
6	37 ^b^	29	39 ^a^	33
7	36 ^b^	28	43 ^a^	29
8	35 ^b^	27	49 ^a^	32
9	31 ^b^	29	49 ^a^	33

The maximum value in each FD column is underlined. Numbers with a different superscript differ significantly from each other.

**Table 3.** FD [%] for different FV threshold values and different number of favourite viewpoints combined from the third session.

**Positions**	**FV threshold value**		

	**40%**	**50%**	**60%**
5	59	56	48
5–7	79	69	66
5-8-10	79	72	72
5-7-8-10	93	86	83
5-7-8-10-13	93	90	83

**Table 4.** Maximum FD and the number of positions needed to reach that FD for different FV threshold values (Th.). Values given for second session (2) and the third session (3).

**Th.**	**Maximum FD (2)**	**Positions needed (2)**	**Maximum FD (3)**	**Positions needed (3)**

**[%]**	**[%]**	**[#]**	**[%]**	**[#]**
5	100	2	97	2
10	93	3	97	2
20	90	3	97	3
30	90	4	97	3
40	83	4	97	4
50	76	5	90	5
60	62	6	86	5
70	59	6	83	7
80	52	5	59	6
90	28	3	41	5
100	7	2	28	4
